# An open-label, randomized, non-inferiority trial of the efficacy and safety of ciprofloxacin versus an aminoglycoside + ciprofloxacin in the treatment of bubonic plague (IMASOY): study protocol for a randomized control trial—an update to the published protocol

**DOI:** 10.1186/s13063-024-08302-7

**Published:** 2024-07-05

**Authors:** Rindra Vatosoa Randremanana, Mihaja Raberahona, Mamy Jean de Dieu Randria, Josephine Bourner, Gabriella Zadonirina, Hanitra Razananaivo, Théodora Mayouya-Gamana, Reziky Mangahasimbola, Elise Pesonel, Annelies Gillesen, Minoarisoa Rajerison, Voahangy Andrianaivoarimanana, Tansy Edwards, Peter Horby, Piero Olliaro

**Affiliations:** 1 Institut Pasteur de Madagascar, Antananarivo, Madagascar; 2 Centre d’Infectiologie Charles Mérieux, University of Antananarivo, Antananarivo, Madagascar; 3 Department of Infectious Diseases, University Hospital Joseph Raseta Befelatanana, Antananarivo, Madagascar; 4 Faculty of Medicine, University of Antananarivo, Antananarivo, Madagascar; 5 ISARIC, Pandemic Sciences Institute, University of Oxford, Oxford, UK; 6 London School of Hygiene and Tropical Medicine, London, UK

## Abstract

This article reports an update to the protocol of the IMASOY trial, which was prospectively registered on clinicaltrials.gov (NCT04110340) in October 2019.

This article reports an update to the protocol of the IMASOY trial (NCT04110340) [1], for which the following major amendments have been made since publication in *Trials*:

## Objectives {7}

### Observational non-comparative study of pneumonic plague

In 2021, new treatment guidelines were released by the US Centers for Disease Control and Prevention that recommend the use of a combination therapy for pneumonic plague. As no clinical equipoise exists to demonstrate a potential benefit of a monotherapy to treat pneumonic plague, it was therefore considered to be unethical to continue randomizing patients with suspected pneumonic plague to receive a monotherapy (ciprofloxacin alone). Patients with pneumonic plague symptoms at admission were therefore enrolled to an observational cohort and the outcomes of the current standard of care treatment (as per the national treatment guidelines) will be reported. Note: patients who are admitted and enrolled with suspected bubonic plague and who later develop pneumonic plague will remain in the randomized cohort, receive appropriate treatment for pneumonic plague as per national treatment guidelines and will be treated as a treatment failure.

### Exploratory objectives

Three exploratory objectives were removed from the protocol. The analysis will not include the following: comparison of different methods for detection of anti-*Yersinia pestis* antibodies; measurement of the performance of qPCR for plague diagnosis; evaluation of new rapid tests.

## Trial design {8}

### Case definitions

The case definition (Table 1) has been modified in line with the revised international plague case definition (2021) [2].

**Table 1:** Case definition for confirmed and probable cases of bubonic and pneumonic plague

	**Confirmed case**	**Probable case**
Bubonic plague	• qPCR is positive on D1 sample or • Culture is positive on D1 sample or • There has been a seroconversion between D1 and D21 samples or a fourfold increase in antibody titre on two separate serological samples between D1 and D21	• RDT (conducted at central laboratory) positive on D1 sample AND • qPCR negative on D1 sample AND • Culture negative on D1 sample AND • No evidence of seroconversion, nor a fourfold increase in antibody titre
Pneumonic plague	[Using sputum samples] • qPCR is positive on D1 sample or • Culture is positive on D1 sample	[Using sputum samples] • RDT (conducted at central laboratory) positive on D1 sample AND • qPCR negative on D1 sample AND • Culture negative on D1 sample

## Intervention description {11a}

### Control arm

Due to the expiration of the national supply of streptomycin in Madagascar and lack of global availability of new stock, the control arm treatment was amended from streptomycin + ciprofloxacin to an aminoglycoside + ciprofloxacin. Under Madagascar’s national treatment guidelines for bubonic plague, gentamicin is the next alternative aminoglycoside treatment option. To account for treatment heterogeneity in the control arm, a sensitivity analysis will be planned as part of the final analysis.

## Outcomes {12}

### Primary endpoint

Following the publication of the results of a study evaluating the precision and intra-rater agreement of the measurement of artificial buboes using a digital calliper [3], it was agreed that the measurement of artificial buboes with a digital calliper is unreliable and raised concerns about the potential impact of significant measurement error on the assessment of the composite primary endpoint of the IMASOY trial—a component of which requires detection of a 25% reduction in bubo size. The primary endpoint was therefore amended to remove component evaluating bubo size. The original composite endpoint was retained as a new secondary endpoint.

## Participant timeline {13}

### Schedule of events

A trial visit was added at M12 (± 2-month window) for patients who return a positive antibody (IgM or IgG) test at M3.

## Trial status

The IMASOY closed to recruitment in March 2024 having recruited over 220 probable and confirmed cases of bubonic plague—the planned minimum sample size was 190. The trial results will be available in Q4 of 2024.

## Data Availability

Not applicable—no data have been collected as part of this publication.
